# The “Third Violin” in the Cytoskeleton Orchestra—The Role of Intermediate Filaments in the Endothelial Cell’s Life

**DOI:** 10.3390/biomedicines10040828

**Published:** 2022-04-01

**Authors:** Anton S. Shakhov, Irina B. Alieva

**Affiliations:** A.N. Belozersky Institute of Physical and Chemical Biology, Lomonosov Moscow State University, 119992 Moscow, Russia; sh.anton90@yandex.ru

**Keywords:** intermediate filaments, vimentin filaments, soluble vimentin, microtubules, actin, plectin, cell contacts, extracellular matrix, inflammation, atherosclerosis

## Abstract

The endothelium plays an important role in the transcytosis of lipoproteins. According to one of the theories, endothelial injury is a triggering factor for the development of atherosclerosis, and intracellular structures, including components of the endotheliocyte cytoskeleton (microtubules, actin, and intermediate filaments), are involved in its development. In contrast to the proteins of tubulin-based microtubules and actin microfilaments, intermediate filaments are comprised of various tissue-specific protein members. Vimentin, the main protein of endothelial intermediate filaments, is one of the most well-studied of these and belongs to type-III intermediate filaments, commonly found in cells of mesenchymal origin. Vimentin filaments are linked mechanically or by signaling molecules to microfilaments and microtubules by which coordinated cell polarisation and migration are carried out, as well as control over several endotheliocyte functions. Moreover, the soluble vimentin acts as an indicator of the state of the cardiovascular system, and the involvement of vimentin in the development and course of atherosclerosis has been demonstrated. Here we discuss current concepts of the participation of vimentin filaments in the vital activity and functioning of endothelial cells, as well as the role of vimentin in the development of inflammatory processes and atherosclerosis.

## 1. Introduction

The development of atherosclerosis and its associated intracellular pathologies have been the focus of attention of numerous researchers over the past hundred years. During this time, the mechanisms of atherogenesis, the processes of endothelial transport, direct and receptor-mediated transcytosis of lipoproteins through the endothelial barrier have been described in detail. The history of atherogenesis research has elicited significant discoveries about the development of the pathological processes involving the vasculature and of the endotheliocyte lining layer. Several theories of pathogenesis have been formulated, one of which proposes that endothelial injury serves as a trigger for the development of atherosclerosis, leading to disruption of lipoprotein transcytosis. After this endothelial impairment, an inflammatory reaction begins resulting in cholesterol accumulation within the disturbed region.

Since the endothelium intracellular structures are directly involved with atherosclerosis, our interest is with components of the tensegrity cytoskeleton (microtubule, actin, and intermediate filaments), which are actively involved with development and progression. Recent research has demonstrated evidence for the functional involvement of vimentin intermediate filaments in the physiological functions of endotheliocyte and the development of inflammatory processes and atherosclerosis.

## 2. Features of Intermediate Filaments—The Third Component of the Cytoskeleton

Historically, during the last 50 years, when the emergence of new molecular-cell approaches and microscopic techniques, marked by the explosive growth of studies on the dynamics and functionality of microtubules and actin filaments, intermediate filaments remained the least studied component of the cell cytoskeleton. The classical concept of the third component of the cytoskeleton [1,2] is as follows: (1) intermediate filaments are located in the form of three-dimensional networks in different parts of the cell cytoplasm, surround the nucleus, participate in the formation of intercellular contacts, and maintain the shape of processes; (2) the main function of intermediate filaments, based on their mechanical properties and self-assembly ability, is to maintain cellular and tissue integrity; (3) intermediate filaments consisting of different proteins are expressed in different types of cells. The latter property distinguishes intermediate filaments from microtubules (polymerized from tubulin) and microfilaments (composed of actin). Indeed, intermediate filaments have a different protein composition, both in different tissues and at different stages of embryonic development and at different stages of differentiation. Moreover, some types of cells simultaneously contain several different intermediate filaments. In total, about 70 genes have been found in the human genome, encoding various proteins of intermediate filaments, which form one of the most numerous protein families. However, since the beginning of the nineties of the last century, interest in the study of intermediate filaments began to actively grow since it turned out that mutations in these proteins are associated with severe skin (keratins), nervous (neurofilaments) human pathologies, including muscular dystrophies (desmin) and cardiomyopathies (desmin and vimentin) [3,4,5,6].

## 3. Classification of Intermediate Filaments and Its Types Found in Endothelial Cells

The homology of protein sequences of intermediate filaments is sometimes no more than 20%; nevertheless, based on biochemical, immunological, and structural similarities, six different types of intermediate filaments are distinguished. According to the modern classification [7], intermediate filaments are divided into keratins type-I (acidic keratins) and keratins type-II (basic keratins) proteins. For the assembly of keratin intermediate filaments, proteins of both types are required, which form heteropolymers. Type-III intermediate filament proteins are comprised of desmin, vimentin, peripherin, and glial acidic proteins. These proteins can form both homopolymers and also heteropolymers with other type-III protein members as well as with the neurofilament light (NF-L) protein. Type-IV intermediate filament proteins are expressed predominantly in nerve cells. This type represents α-internexin and a triplet of neurofilament proteins: NF-L, NF-M, and NF-H (neurofilament light, medium, and heavy proteins, respectively). The protein nestin, first discovered in the precursors of nerve cells, is sometimes referred to as a special type of intermediate filament protein. However, based on its structural features, nestin can be classified as type IV. Type-V intermediate filament proteins include nuclear lamins, and type-VI proteins include two proteins found in the lens of the eye (filensin (CP115) and phakinin (CP49)). Keratin intermediate filaments (53–55 kDa) are characteristic of epithelial cells. Desmin intermediate filaments of muscle tissue (with the exception of vascular myocytes) are composed of desmin protein (53–55 kDa). Vimentin filaments, characteristic of various cells of mesenchymal origin (fibroblasts, macrophages, osteoblasts, endothelium, and vascular smooth myocytes), consist of the protein vimentin (54–58 kDa). Peripherin (57 kDa) is present in peripheral neurons, participating in the assembly of intermediate filaments in place with neurofilament proteins. Neurofilaments are intermediate filaments of neurons that play an important role in maintaining the shape of the processes of nerve cells. They consist of at least three high molecular weight polypeptides (68, 140, and 210 kDa). Glial filaments contain glial fibrillar acidic protein (56–58 kDa) and are found only in glial cells (astrocytes, oligodendrocytes).

Proteins, which are also referred to as proteins of intermediate filaments, are present not only in the cytoplasm (as listed above) but also in the cell nucleus. The dense envelope located under the nuclear membrane, imparting rigidity to the nucleus (lamina), consists of lamina proteins. Lamins are intermediate filaments of nuclei of various types of cells that form an intranuclear skeleton (karyoskeleton). Unlike other intermediate filament proteins, laminae do not form filaments but rather a reticular structure. Even with the destruction of cell membranes (for example, treatment with detergents), the nucleus retains its integrity due to the lamina.

There are several types of intermediate filaments present in endothelial cells. The main protein of intermediate filaments in the endothelium is vimentin [8,9]. The structure of vimentin is conserved in mammals and shows dynamic expression profiles in various cell types and different developmental stages. Additionally, nestin was found in endotheliocytes [10,11,12] as well as neurofilaments [13]. Nestin has been found in cardiac endothelial cells and is generally considered a marker of revascularization [10,11,12]. In addition, nestin expression is specific not only for proliferating endothelial cells (bovine aortic endothelial cells (BAEC) in vitro), but in the vascular endothelium of brain tumors in the stage of rapid growth [14]. Furthermore, the rate of nestin expression is one of the important criteria for prognosis in breast cancer [15]. After studying nestin-positive microvascular density in breast cancer patients, Nowak and her colleagues [15] concluded that a high level of nestin expression is characteristic of newly formed tumor vessels. In addition, a high level of nestin expression may be related to an aggressive course of the disease and a poorer prognosis. In contrast, it is necessary to mention another nestin-related research work [16], which included body-wide transcriptome and protein-profiling analysis. Dusart and colleagues demonstrated, that nestin is constitutively expressed in human endothelial cells; its expression does not depend on cell proliferative status and is not specific to tumor endothelium. Nestin co-localizes with vimentin in different types of endothelia in vitro (umbilical vein (HUVEC), dermal microvessels (HDMEC), the coronary artery (HCAEC), and the pulmonary artery (HPAEC) under static conditions and laminar shear stress [16]. Especially interesting is that nestin expression is lower in regions of atherosclerotic plaques than in normal vessels [16].

As noted earlier, neurofilaments are the type-IV family of intermediate filaments and are usually associated with neural tissues. Nestin (member of type-VI intermediate filament), is a well-known marker of endothelial cells in newly formed blood vessels and is developmentally and structurally related to type-IV intermediate filaments. Based on this similarity, Rusu and colleagues [13] noted the neurofilament positive labeling of endothelial cells may be due to interactions of nestin and neurofilaments within cadaver samples (sinoatrial nodes/right atrial walls) of both normal and diabetic donors [11]. Positive labeling of endothelial cells leads to the question if neurofilaments may qualify as markers of angiogenesis [13].

## 4. Molecular Structure of Vimentin Filaments as the Basis of Their Remarkable Mechanical Properties

The most conservative representation of type-III filaments is with vimentin. The outstanding mechanical properties of vimentin filaments are explained by the subunit packing geometry making up the filament. The vimentin monomer is subdivided into three domains: N-terminal (“head”), C-terminal (“tail”), and a highly conserved central domain, which includes four coiled domains and three linker non-coiled regions [17]. The central domain contains tandem repeats of amino acids (heptade repeats), which are capable of forming a supercoiled dimer. The vimentin dimer, the basic structural element of the vimentin filament, consists of 466 amino acid residues [18]. The two dimers bind anti-parallel and stepwise, forming a tetramer, the fundamental unit of intermediate filaments (ULF) [16].

As a result, intermediate filaments have unique stability against applied forces and mechanical rupture. This property depends on the formation of many simultaneous bonds along the filament diameter, many of which are electrostatic or hydrogen bonds. In actin (microfilaments) or tubulin polymers (microtubules), the binding sites between subunits are largely hydrophobic. In contrast, the bonds between vimentin dimers in the tetramer (and amongst tetramer filaments) involve the overlap of local regions of opposing charge. These interactions occur not only in regions within the filament core, but the alpha helices, and around the N-terminus of the proteins [19,20].

## 5. Vimentin Filaments: The Role of Post-Translational Modifications in the Regulation of Depolymerization and Stabilization of Vimentin Filaments

After synthesis, intermediate filaments can undergo various chemical modifications [21]. Regulation of the vimentin network is highly complex and is driven by post-translational modifications, such as phosphorylation and cleavage by intracellular proteases. The reorganization of the network of vimentin filaments occurs through phosphorylation, a post-translational modification, due to which the assembly and disassembly of filaments occur during the cell cycle [22]. The first evidence of the dependence of vimentin organization on phosphorylation was obtained in in vitro experiments on complete disassembly of vimentin filaments using purified protein kinase C or cAMP-dependent protein kinase [23]. Analysis of vimentin mutants with deletions at the N-terminus (“head”) revealed that it is the N-terminal domain that is critical for the assembly of vimentin filaments, as well as for the formation of the network [24]. Of the many found vimentin phosphorylation sites (serine residues), those responsible for filament disassembly are located precisely on the vimentin head domain and are phosphorylated by a number of protein kinases. Phosphorylation of the N-terminal domain increases the distance between the two head domains of the dimer, which makes it impossible to form a complete filament from vimentin tetramers [7]. Vimentin also undergoes other post-translational modifications, such as sumoylation, citrullination, and glycosylation by O-linked N-acetylglucosamine (O-GlcNAcylation) [7]. In the process of apoptosis, arginine deamination (citrullination) of vimentin occurs [25], and citrullination sites are located in the “head” domain [22]. This modification occurs in the presence of calcium ions and causes depolymerization of vimentin filaments. O-N-acetylglucosamine glycosylation (O-GlcNAcylation) of vimentin in neurons can prevent excessive phosphorylation of filaments and their depolymerization [26]. Vimentin sumoylation is triggered by STAT inhibition in glioblastoma cells [27]. All this indicates the importance of post-translational modifications in the regulation of depolymerization and stabilization of vimentin filaments.

## 6. Features of Intracellular Functions in the Endothelium and Dynamics of Vimentin Filaments

While the functions of microtubules and actin filaments were studied by changing their dynamics or in disruption/recovery experiments, any drugs/small molecules for the selective targeting of vimentin (or any other intermediate filaments) were not available until now. Hereby, the mechanistic understanding of this cytoskeletal component is strongly limited. From the other side, the first global vimentin knockout mouse generated more then 25 years ago was described as having no phenotype [28], which contradicted the fact of extreme evolutionary conservation of vimentin in vertebrates [29], leading to the false conclusion of its little physiological significance. However, defects in the Vim −/− mice on the cellular-level cause impairments in normal physiological processes, including organ development [30], angiogenesis [31], vascular stiffness [32], and many others. Summarizing the results of global Vim −/− mice studies, the real function of vimentin lays at the organismal level of cells and is important under both physiological and pathophysiological stress conditions.

However, since it was shown that vimentin filaments are the main intermediate filaments in endothelial cells [8], these components of the cytoskeleton have been extensively studied in endothelial cells of various vessels. It turned out that the amount of vimentin in endotheliocytes of different parts of the circulatory system varies greatly depending on the hemodynamic load upon the cell; in the endotheliocytes of the left ventricle of the heart and aorta there is more vimentin compared to the superior vena cava, atrium, and right ventricle [33]. At the same time, it turned out that vimentin in the endothelium in vitro is always significantly higher than in endotheliocytes isolated from any organ [31]. The data obtained indicate that one of the main functions of vimentin filaments in the endothelial cell is mechanical. Vimentin preserves cell morphology and resists stretching and compression of the vessel under the influence of blood flow; thus, it is an important factor in maintaining tissue integrity [7,33,34,35].

For a long time, intermediate filaments were considered stable structures due to their insoluble nature [34,36]. Then, using GFP-vimentin, information was obtained that vimentin filaments are capable of rearrangements and can elongate and shorten even with a relatively stable cell shape [37]. As a result, the vimentin filament network, originally described as a stable “skeletal” element of cells, was revealed by live-cell-imaging studies to be a very dynamic system—FRAP (fluorescence recovery after photobleaching) studies demonstrated active recovery of vimentin fluorescence in fibroblast cytoplasm after photobleaching [37]. It turned out that vimentin had a recovery half-time of 5 ± 3 min [37], exhibiting dynamic properties similar to those of microtubules [38] and actin filaments [39].

Vimentin filaments are highly dynamic in endothelial cells. In experiments on bovine endothelium, Helmke and colleagues [40] found that when exposed to a fluid flow, vimentin filaments undergo rapid changes—a change in the position of some parts of the vimentin network was observed. However, at the same time, a sharp restructuring of the vimentin network in the endotheliocyte does not occur [40]. Subsequently, it was demonstrated in vitro on the endothelial monolayer that a more pronounced rearrangement of the network of vimentin filaments occurs above the nucleus as well as behind it (in relation to the fluid flow acting on the cell) [41]. It was also found that similar changes in the network of vimentin filaments occurred in the marginal regions of neighboring (contacting) cells [41]. As a result, to date, a high dynamism of vimentin filaments and their association with the molecular motors of dynein and kinesin have been demonstrated [42,43]. Assembly, stabilization, and disassembly of vimentin filaments immediately occur under the influence of various exogenous stimuli [7]. Sharp changes in the structure of vimentin filaments occur when the endothelium is exposed to hypoxia; after an hour of hypoxia, an elongation of the vimentin network was observed, and the proportion of insoluble vimentin also increased [9]. Moreover, hypoxia induces changes in the phosphorylation of vimentin and affects the activity of PAK1 kinase, which is responsible for the assembly of vimentin filaments. It turned out that the dynamics of vimentin filaments is directly involved in the implementation of the main barrier function of the endothelium—inhibition of vimentin polymerization increases the permeability of the endothelial monolayer [9].

## 7. Linker Proteins—The Connection of Vimentin Filaments with Other Cellular Structures

Proteins of particular interest which can bind to vimentin are plectin and nesprin-3. In the animal cell, the network of vimentin filaments is actively reorganized using plectin [44]. It has also been shown that vimentin is able to directly bind to nesprin-3 [45]. Nesprin-3 is actively expressed in HAEC cells and is localized in the nuclear envelope [45]. It is believed that nesprin-3 is the link between the nuclear membrane and the plasma membrane. Nesprin-3 regulates the shape of the cell; knockdown of the nesprin-3 gene causes a noticeable elongation of the cell. Nesprin-3 not only affects the perinuclear organization of the cytoskeleton, it is required to attract the centrosome to the region of the nuclear envelope. It is possible that nesprin-3 is a scaffold for plectin, providing its perinuclear organization; it is also necessary for the connection of the centrosome with the nucleus. Nesprin-3 is also required for centrosome polarization caused by fluid flow and flow-induced migration of endothelial cells. The cell center is located in a network of vimentin intermediate filaments, held together by plectin stitches. In the absence of nesprin-3, the perinuclear network disappears, and the centrosome in this state can be associated with elements of the cytoskeleton far from the nucleus. Thus, nesprin-3 regulates the perinuclear architecture of the endothelial cytoskeleton, cell shape, and is also involved in the mechanism of flow-induced mechanotransduction.

Fragmentary information, suggesting a possible connection between intermediate filaments and the centrosome, appeared at the end of the last century. It was shown that after treatment with nocodazole, the rearrangement of vimentin intermediate filaments in the pericentrosomal region occurred, but, at the same time, their direct interaction was not found [46]. Later, using electron microscopy, foci of convergence of intermediate filaments, representing dense granules with a diameter of about 0.25 µm, were found in fibroblasts near the centrosome [47]. These data suggested that the centrosome can serve as a center for the organization of intermediate filaments and be responsible for their distribution over the cytoplasm [47]. Then, a hypothesis was put forward, according to which the sites of attachment of vimentin filaments may be located in the pericentriolar material [48]. These sites were located in the centrosome independently of microtubules; disturbances in the organization of microtubules did not affect the location of the foci of convergence of vimentin filaments [48]. Later it became known that the plectin protein might be involved in the interaction of vimentin filaments and the positioning of the centrosome in endothelial cells [45]. Plectin is able to interact with the BRCA1 protein and form a complex that controls the position of the centrosome [49]. According to the model [45], there is a network of vimentin filaments around the centrosome connected to each other by plectin, the perinuclear localization of which is mediated by the nesprin-3 ligament protein. This network is dynamic—under the action of fluid flow, the centrosome can be detached from the nucleus and its displacement [45].

However, the question of whether the centrosome is capable of organizing intermediate filaments similarly to microtubules remains open. Relatively recently, it was shown that not only microtubules and actin filaments interact with each other, but also vimentin filaments are able to bind to both microtubules and actin filaments [49]. Communication can be carried out at the regulatory level—transport of precursors of vimentin filaments is under the control of actin filaments and regulatory kinases p21 and Rho [50] and occurs along microtubules, while the process does not depend on the dynamics of microtubules [51]. It turned out that the aforementioned linker protein, plectin, plays an important role in creating an ordered vimentin network in the cell [52]. The link between vimentin and actin filaments, mediated by plectin, is extremely important for cellular morphogenesis [53]. Apparently, plectin also contributes to maintaining the integrity of the endothelial monolayer—it is able to regulate the interaction of the vimentin network and actin filaments [54]. During the cultivation of plectin-deficient cells, large breaks in the endothelial monolayer were observed, which could probably be associated with a disruption in the connection of actin filaments with VE-cadherin contacts [54]. It was shown that plectin deficiency leads to the (1) vimentin breaking; (2) severe distortions of adherens junctions and tight junctions; (3) upregulation of actin stress fibers. As a result, cellular contractility of endothelial cells cytoskeleton increased, and plectin-deficient endothelial cell monolayers were leakier and demonstrated reduction of mechanical resilience in fluid-shear stress and mechanical stretch conditions. Summarizing their results, the authors put forward an idea that adherens junctions defects and actin stress fibers upregulation in plectin-deficient cells are rooted in vimentin cytoskeleton perturbations, precisely because disruption of vimentin filaments by itself could mimic very similar phenotypes in wild-type cells. Similar effects were observed in vivo in the experiments with endothelium-restricted conditional plectin-knockout mice [54]. Numerous studies support that vimentin can regulate focal adhesions, and mature focal adhesions serve as docking sites for vimentin filaments [55].

Vimentin directly binds the tail of b3- [56] and a2b1-integrins [57], and GFAP and vimentin interact with adhesion molecules, such as vinculin and talin [58].

Focal adhesions link integrins to vimentin intermediate filaments and actin microfilaments; these interactions are carried out through plectin, a protein that cross-links microtubules, microfilaments, and intermediate filaments [59].

Although intermediate filaments have unique mechanical properties, they need to be coupled with microtubules to maintain cell shape. During the experimental destruction of microtubules, bundles of vimentin filaments are concentrated around the nucleus—their “collapse” occurs. Numerous studies of recent years have shown that vimentin filaments pattern microtubules during directed migration [60] and template microtubule networks to enhance persistence during cell polarization and directed migration [61]. It is supposed now that vimentin promotes cell migration by modulating the dynamics of microtubules and the actomyosin network [60].

**Figure 1:**
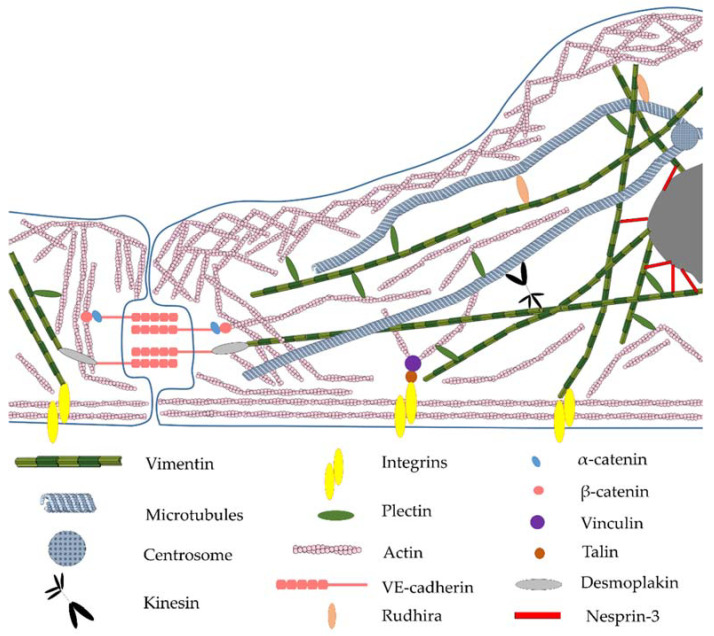
Scheme of interconnection of vimentin and other cytoskeleton components and endothelial cell proteins.

Thus, all three components of the cytoskeleton work in the endothelial cell interconnected [62], both at the structural (Figure 1) and functional levels, ensuring the normal vital activity of the endothelium and the performance of the barrier function [63,64,65,66]. Possibly, in the endothelial cells, vimentin can interact differently with β- and γ-isoforms of nonmuscle actin [67].

## 8. The Role of Vimentin in Angiogenesis, the Development of Inflammatory Processes and Vascular Pathologies

For the first time, the involvement of vimentin in angiogenesis was shown by Eckes et al., who reported that vimentin silencing in mice led to a lag in granulation tissue formation, suggesting a link between vimentin and angiogenesis [68]. Later in a few investigations, the role of vimentin in angiogenesis was experimentally confirmed. Recent research results suggest that vimentin is an integral regulator of endothelial sprouting; vimentin complexed with focal adhesion kinase, regulate (1) focal adhesion kinase expression, (2) focal adhesion organization, and (3) adhesion in endothelial cells, which are key events involved in angiogenic sprouting [7,69,70].

Vimentin is one of the most important links in the regulation of Notch ligands during angiogenesis [31]. Vimentin interacts with the jagged ligand, which is necessary for Notch-activating trans-endocytosis, receptor binding, and then Notch activation [31]. Moreover, vimentin can regulate jagged recycling and levels of that ligand on the cell membrane [31]. Vimentin deletion destabilizes sprouting angiogenesis and disrupts embryonic angiogenesis [31].

A recent study has demonstrated the importance of protein linker Rudhira in the cytoskeleton crosstalk for cell motility and accurate performing of angiogenesis [71]. Rudhira protein directly interacts and couples vimentin filaments and microtubules in endothelial cells. BCAS3 domain of Rudhira is necessary for vimentin-microtubules association and cell migration [71].

Initially, vimentin is known as an intracellular protein. In the early 2000s, it was discovered [72] that the widely used vimentin marker PAL-E detects a secretory form of vimentin (PAL-E-reactive vimentin) without binding to intermediate filament vimentin. It turned out that PAL-E-reactive vimentin is found in the circulating blood as it is secreted extracellularly by endothelial cells of blood vessels and activated macrophages [72]. The authors of the study demonstrated that the secretory vimentin is not a translation product of endothelium-specific mRNA, and, most likely, undergoes post-translational modifications [72]. Today it is known that increased serum levels of secretory vimentin are associated with the presence and severity of coronary artery disease; further, vimentin level is an independent determinant of this disease [73]. In vitro secretory vimentin can play a role of inflammatory trigger and promote macrophage-endothelial cell adhesion [73]. Moreover, secretory vimentin protein provokes atherogenesis in ApoE−/− mice in vivo [73]. The biological role of extracellular vimentin is currently unknown [71], especially detailed aspects of its excretion and function [74].

An increased level of vimentin was observed in the sites of atherosclerotic lesions, which, according to some data, directly leads to the endothelial-to-mesenchymal transition in these areas [75]. Vimentin-null mice show a reduction of ability to remodeling arteries and enlarged stiffness, contractility, and endothelial dysfunction [32]. Vimentin deficiency in mice cause an increased expression of subendothelial basement membrane components [32] and probably stimulate phenotypic alterations and endothelial-to-mesenchymal transition [76]. In general, the role of vimentin in the endothelial-to-mesenchymal transition is being studied, especially active in recent years precisely in connection with diseases of the cardiovascular system [76]. A number of very interesting works have been devoted to vimentin involvement in vascular diseases, including pulmonary arterial hypertension. It was especially shown that vimentin phosphorylation is one of the main stages of endothelial-to-mesenchymal transition in pulmonary hypertension [77,78,79].

On the other hand, it is known that microRNA miR-144 may be a regulator of atherosclerosis development via changes in vimentin signaling [80]. miR144 targets vimentin in vitro and in vivo [80]. miR-144 mice display increased secretory vimentin and are more sensitive to atherosclerotic processes, and accumulated vimentin in the knockout mice aorta strengthen atherosclerotic plaque formation [80].

Vimentin, which expressed on the surface of endothelial cells, plays a crucial role in lymphocyte adhesion and transmigration through the endothelium [81]. The integrity of the endothelial monolayer was compromised in vimentin-null mice, and vimentin deficiency resulted in the incorrect positioning of homing molecules [81].

It should be noted that the exact role of vimentin in inflammation is not entirely clear, but the role of actin is revealed in much more detail [82]. However, vimentin can bind to N-acetylglucosamine [83], a moiety on PSGL-1 (P-selectin glycoprotein ligand-1) on leukocytes [84]. Moreover, vimentin, which is expressed on the cell surface of endothelium [85], can bind to soluble CD44, the soluble form of E-selectin ligand found on neutrophils [86]. Lam, with colleagues [87], have managed to prevent the initial stage of acute inflammatory response using recombinant human vimentin (rhVim). Leukocyte adhesion to endothelial and platelet monolayers decreased after treatment of rhVim, which bind specifically to P-selectin, not to PSGL-1 [87].

Da and colleagues [88] demonstrated that vimentin on the platelet surface binds to the A2 domain within the activated form of von Willebrand Factor. Lately, it was shown that extracellular vimentin essentially contributes to von Willebrand Factor string formation via A2 domain binding [89]. Pharmacologically targeting the vimentin/VWF von Willebrand Factor interaction through the A2 domain can promote improved reperfusion after ischemic stroke [89].

Thus, vimentin, both in the form of filaments and in a soluble form, is one of the most important links in the molecular processes observed during the course of vascular diseases, atherosclerosis, etc. (Table 1).

## 9. Conclusions and Perspectives

Summarizing the data listed above, it should be concluded that over the past five years, our knowledge about the involvement of vimentin into the vital activity and functioning of endothelial cells, as well as in the development of angiogenetic, inflammatory, and vascular pathologies has expanded significantly.

The main function of cell cytoskeleton intermediate filaments has long been considered to be the maintenance of cellular and tissue integrity due to their mechanical properties. In addition, intermediate filaments play an important role in the distribution of proteins and organelles throughout the cell. There has been significant recent progress in understanding the cellular function of intermediate filaments, including their role within the endothelial cell. It has turned out that intermediate filaments are not static structures in their mechanical support (as previously thought), but they interact functionally with other components of the cytoskeleton and other cellular processes. In many experimental works, the involvement of vimentin in plenty of cellular processes has been demonstrated. Vimentin is also involved in the processes of intercellular exchange due to its connection with the extracellular matrix. It has recently been found that extracellular vimentin plays a role of attachment factor for SARS-CoV-2 on human endothelial cells; vimentin binds to the SARS-CoV-2 spike protein [90]. shRNA-mediated knockdown of vimentin reduced SARS-CoV-2 infection of human endothelial cells [90].

This review summarizes the experimental data related to the intracellular connections and functions for vimentin in the endothelial cells as well as vimentin involvement in angiogenesis. Vimentin equilibrates Notch-signaling activities and indirectly controls signaling strength [31]. These facts may stimulate the research of Notch pathway components and vimentin in other cell systems in which these molecules may co-express. Another obvious mainstream of research is finding out the role of vimentin in inflammation. The further search for linker proteins and common signaling pathways that synchronize the interaction of vimentin filaments with microtubules and actin microfilaments also looks promising. Perspectives of future studies are connecting to determine how vimentin might control and coordinate the mechanical, spatial, and biochemical properties of endothelial cells critical for specific endothelial functions, including the maintenance of the endothelial barrier, normal and pathological angiogenesis, de novo vascularization, endothelial-to-mesenchymal transition, and their involvement in the development of inflammation, ischemic stroke, and coronary disease.

## Figures and Tables

**Table 1:** Vimentin in the development of angiogenetic, inflammatory, and vascular pathologies.

Pathological Processes	Role of Vimentin	References
Disturbances of angiogenesis and cell motility	Increased vimentin level in the sites of atherosclerotic lesions leads to the endothelial-to-mesenchymal transition Vimentin deletion destabilizes sprouting angiogenesis and disrupts embryonic angiogenesis	Evrard et al., 2016 [75] Antfolk et al., 2017 [31]
	Importance of protein linker Rudhira for vimentin-microtubules association and angiogenesis	Joshi and Inamdar, 2019 [71]
	Secretory vimentin provoke atherogenesis in Apolipoprotein E (ApoE−/−) deficient mice in vivo	Gong et al., 2019 [73]
Inflammation	Prevention of the initial stage of acute inflammatory response using recombinant human vimentin	Lam et al., 2018 [87]
	Secretory vimentin as an inflammatory trigger (promotion of macrophage-endothelial cell adhesion)	Gong et al., 2019 [73]
Vascular diseases	Reduction of ability to remodeling arteries and enlarged stiffness, contractility and endothelial dysfunction in vimentin-null mice	Langlois et al., 2017 [32]
	Targeting the vimentin/VWF von Willebrand Factor interaction promote improved reperfusion after ischemic stroke	Fasipe et al., 2018 [89]
	Increased serum levels of secretory vimentin are associated with the presence and severity of coronary artery disease	Gong et al., 2019 [73]
	Vimentin accumulation in the knockout (miR-144) mice aorta strengthen atherosclerotic plaque formation	He et al., 2020 [80]

## Data Availability

Not applicable.
